# Eight‐color multiparameter flow cytometry (EuroFlow‐NGF) is as sensitive as next‐generation sequencing in detecting minimal/measurable residual disease in autografts of patients with multiple myeloma

**DOI:** 10.1002/jha2.633

**Published:** 2023-01-24

**Authors:** Ryota Urushihara, Naoki Takezako, Takeshi Yoroidaka, Takeshi Yamashita, Ryoichi Murata, Kenji Satou, Shinji Nakao, Hiroyuki Takamatsu

**Affiliations:** ^1^ Department of Hematology, Faculty of Medicine, Institute of Medical, Pharmaceutical and Health Sciences Kanazawa University Kanazawa Japan; ^2^ Faculty of Transdisciplinary Sciences for Innovation Institute of Transdisciplinary Sciences for Innovation Kanazawa University Kanazawa Japan; ^3^ Department of Hematology Disaster Medical Center of Japan Tachikawa Japan; ^4^ Division of Internal Medicine Keiju Kanazawa Hospital Kanazawa Japan

**Keywords:** autologous peripheral blood stem‐cell transplantation, minimal/measurable residual disease (MRD), multiparameter flow cytometry, myeloma, next‐generation sequencing

## Abstract

The prognostic value of minimal/measurable residual disease (MRD) detection in autografts of patients with multiple myeloma (MM) in an autologous stem‐cell transplantation setting has been reported. Next‐generation flow (NGF) cytometry has lower sensitivity (2 × 10^−6^) to detect MRD than next‐generation sequencing (NGS) (<10^−6^). We compared the clinical value of high‐sensitivity NGF (cutoff: <10^−6^) and NGS (cutoff: 10^−6^) for the detection of MRD in the cryopreserved autografts of 49 patients with newly diagnosed MM. The sensitivity test using frozen/thawed autografts revealed a strong correlation among MRD levels of 5 × 10^−7^ and 1 × 10^−4^ (*r* = 0.9997, *p* < 0.0001) when an adequate number of cells were analyzed. Autograft MRD levels determined using NGF and NGS were highly correlated (*r* = 0.811, *p* < 0.0001). MRD‐negative patients identified with NGF (cutoff: <10^−6^) showed significantly longer progression‐free survival (PFS) than MRD‐positive patients (*p* = 0.026). The PFS of MRD‐negative patients determined by NGS (cutoff: 10^−6^) was similar to that determined by NGF. These results show that the high‐sensitivity NGF method can assess MRD in frozen/thawed autografts, and its prognostic value is comparable to that of NGS.

## 1 INTRODUCTION

Although various novel agents have been developed for the treatment of multiple myeloma (MM), autologous stem‐cell transplantation (ASCT) remains the gold‐standard treatment for MM [1, 2, 3, 4]. Tandem ASCT has recently been recommended for patients with MM with high‐risk chromosomal abnormalities [5]. However, autografts for ASCT often contain myeloma cells, which can lead to MM relapse. Treatment with novel agents can yield deeper responses, such as minimal/measurable residual disease (MRD)‐negative status, than conventional therapies, and allow autografts to be free of MM cells. It has been reported that this contamination issue was solved in autografts with an MRD level of <10^−5^ [6], or <10^−6^ [7] in selected patients, and the prognosis of patients who received MRD‐negative autografts was significantly better than those who received MRD‐positive ones. In this regard, the development of methods for assessing extremely deep responses in MM is important.

The prognostic value of MRD detection in autografts in an ASCT setting has been reported using EuroFlow next‐generation flow (NGF) cytometry and next‐generation sequencing (NGS) [8, 9]. The main limitation of NGF is that it has a lower sensitivity (2 × 10^−6^) than NGS (<10^−6^) due to the limited number of cells in the bone marrow (BM) available for NGF. However, the sensitivity for detecting MRD may increase by analyzing larger numbers of cells in the autografts. To test this hypothesis, we increased the number of mononuclear cells examined by NGF to 8 × 10^7^ to detect MRD in autografts, and we compared the prognostic value of detected MRD between NGF and NGS.

## 2 PATIENTS AND METHODS

Forty‐nine patients who had been newly diagnosed with MM at the National Hospital Organization Disaster Medical Center, Kanazawa University Hospital, or Keiju Kanazawa Hospital were analyzed in this study (Table 1). All patients received bortezomib (Bort)‐based chemotherapy for induction, followed by high‐dose melphalan (200 mg/m^2^) for ASCT preconditioning. Eight patients received consolidation therapy with carfilzomib–lenalidomide (Len)–dexamethasone (Dex) (*n* = 3), Bort–Len–Dex (*n* = 1), Bort–thalidomide‐Dex (*n* = 2), or Len–Dex (*n* = 2), and 44 (90%) patients received maintenance treatment with Len (*n* = 39), thalidomide (*n* = 1), Bort (*n* = 3), or thalidomide and Len (*n* = 1). ASCT was performed between August 2009 and December 2017, with a data cutoff of December 31, 2020.

**TABLE 1:** Patient characteristics

Parameter	Patients (*n* = 49)
Age at ASCT, years (range)	59 (41–68)
Sex	
Male/female, no. (%)	26 (53)/23 (47)
M‐protein type, no. (%)	
IgG/IgA/IgD/light‐chain only/nonsecretory	33 (67)/4 (8)/1 (2)/10 (20)/1 (2)
*κ*/*λ*	31 (63)/17 (35)
ISS stage at diagnosis, no. (%)	
I/II/III	12 (24)/25 (51) 12 (24)
R‐ISS stage at diagnosis, no. (%)	
I/II/III/NA	3 (6)/28 (57)/6 (12)/12 (24)
High‐risk chromosomal abnormality^a^, no. (%)	15 (31)
Cytogenetics based on G‐banding, no. (%)	
Normal karyotype	40 (82)
Hypodiploid (≤44)	1 (2)
Pseudodiploid (45, 46 with abnormalities)	6 (12)
Hyperdiploid (47–74)	1 (2)
Not assessable	1 (2)
Complex	2 (4)
13q‐/monosomy 13	2 (4)
Induction chemotherapy, no. (%)	
BD/VCD/PAD/VRD/VTD	27 (55)/4 (8)/16 (33)/1 (2)/1 (2)
Autografts mobilization regimen, no. (%)	
CY+G‐CSF/G‐CSF/EDAP+G‐CSF/Bort+CY+G‐CSF	15 (31)/7 (14)/26 (53)/1 (2)
HDT Mel 200 mg/m^2^, no. (%)	49 (100)
plus Bort+Dex	28 (57)
Single/tandem	48 (98)/1 (2)
Post‐ASCT therapy using novel agent, no. (%)	44 (90)
Bort therapy/IMiDs therapy/PIs+IMiDs therapy	2 (4)/33 (67)/9 (18)
Pre‐ASCT response, no. (%)	
sCR+CR/VGPR/PR/SD/NA	3 (6)/15 (31)/20 (41)/1 (2)/10 (20)
Best response, no. (%)	
sCR/CR/VGPR/PR	33 (67)/2 (4)/12 (24)/2 (4)

Abbreviations: ASCT, autologous stem‐cell transplantation; BD, bortezomib plus dexamethasone; Bort, bortezomib; CR, complete response; CY, cyclophosphamide; Dex, dexamethasone; EDAP, etoposide, dexamethasone, cytarabine, and cisplatin; G‐CSF, granulocyte colony‐stimulating factor; HDT, high‐dose therapy; IMiDs, immunomodulatory drugs (lenalidomide/thalidomide); Pis, proteasome inhibitors (bortezomib/carfilzomib); ISS, International Staging System; Mel, melphalan; NA, not assessed; PAD, bortezomib and doxorubicin, and dexamethasone; PR, partial response; R‐ISS, revised ISS; sCR, stringent complete response; SD, stable disease; VCD, bortezomib and cyclophosphamide, and dexamethasone; VGPR, very good partial response; VRD, bortezomib and lenalidomide, and dexamethasone; VTD, bortezomib and thalidomide, and dexamethasone.

^a^ *t*(4;14)/*t*(14;16)/del 17p by iFISH; *t*(4;14) (*n* = 10), *t*(14;16) (*n* = 1), del17p (*n* = 2); and *t*(4;14) and del 17p (*n* = 2).

We performed NGS and NGF analyses of each cryopreserved autograft to increase the sensitivity of MRD detection. Frozen autografts (*n* = 57; prognosis analysis, *n* = 49; comparison between fresh and thawed frozen autografts, *n* = 8) and primary myeloma cells (*n* = 1) were thawed for MRD assessment using NGF and/or NGS. To compare the MRD using NGF between fresh and thawed autografts, we used a 1‐ml aliquot of each fresh autograft; the same amount of aliquot was frozen and later thawed for the MRD assessments. CD138^+^ myeloma cells derived from frozen primary myeloma cells were isolated using EasySep^TM^ Human CD138 Positive Selection Kit II (ST‐17877; STEMCELL Technologies, Vancouver, BC, Canada), according to the manufacturer's instructions.

All patients were treated using institutional review board–approved protocols or standard treatment protocols and provided consent in accordance with the Declaration of Helsinki. This study was approved by the Institutional Review Board of Kanazawa University (No. 2015‐231) and each hospital.

### 2.1 MRD detection by NGS

NGS‐based MRD assessment was performed using Adaptive's standardized NGS–MRD assay (Seattle, WA, USA) [10].

### 2.2 Cryopreservation and thawing of autografts and MRD detection by NGF

Autografts derived from patients with MM were preserved by adding a mixture of CP‐1 High Grade (Kyokuto Pharmaceutical Industrial Co., Ltd) plus 25% albumin (32:68 volume ratio), of the same volume of the autografts, at −80°C until MRD assessment. The preserved autografts were thawed at 37°C in a water bath and washed once with RPMI1640 plus 10% FCS. Debris derived from dead cells was removed using a 40‐μm mesh filter (Falcon Cell Strainer #355340; Corning Inc., Corning, NY, USA). The thawed cell suspension was processed according to section 2.5 of the sample processing protocol for the EuroFlow Bulk Lysis protocol for MRD panels (version 1.1 [May 6, 2014]). We used the EuroFlow‐NGF method described in a previous report [11] and modified to increase the sensitivity of MRD detection by capturing up to 8 × 10^7^ cells of autografts as described in the Supporting Information section.

### 2.3 Statistical analysis

Concordance was analyzed using Pearson's coefficient test. For the evaluation of correlations between the MRD levels determined using NGF and NGS, a logarithmic transformation of the data was performed. Progression‐free survival (PFS) was defined as survival from ASCT until disease progression [12] or death from any cause. PFS and overall survival (OS) were calculated from the time of ASCT until the date of death, by any cause, or the date of last contact. Survival curves were plotted using the Kaplan–Meier method, and the log‐rank test was used for comparisons between groups. Cox proportional hazard analysis with Firth's penalized (partial) likelihood was used to calculate the hazard ratios with the 95% confidence intervals for each variable [13]. All statistical analyses were performed using EZR software package (Saitama Medical Center, Jichi Medical University, Saitama, Japan) [14]. Statistical significance was set at *p* < 0.05.

## 3 RESULTS

### 3.1 Difference in the MRD between fresh and frozen/thawed autografts detected by NGF

**FIGURE 1:**
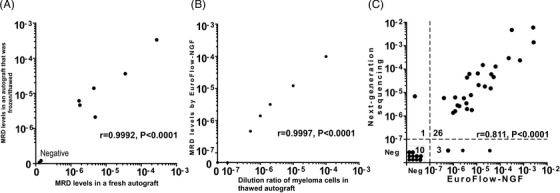
**Detection of minimal/measurable residual disease (MRD) in thawed frozen autografts by EuroFlow next‐generation‐flow (NGF) and next‐generation‐sequencing (NGS)**. (A) Comparison of MRD levels determined by NGF in fresh and thawed autografts. (B) Calibration of MRD levels determined by NGF in serially diluted myeloma cells in autografts. (C) Comparison of MRD detection by NGS and NGF in autografts. Concordance was analyzed using Pearson's coefficient test.

To determine whether cryopreservation of autografts affects the results of MRD detection using NGF, both fresh and cryopreserved autograft samples from eight patients with MM were analyzed with NGF. MRD levels in the fresh and thawed frozen autografts detected by NGF showed strong positive correlation (Figure 1A; *r* = 0.9992, *p* < 0.0001). We thus used thawed frozen autografts to assess MRD levels in the subsequent analyses.

### 3.2 MRD sensitivity test using dilution series of thawed frozen primary MM cells and autografts

We then determined the sensitivity of NGF for detecting MRD using a dilution series of thawed frozen CD138^+^ primary MM cells in a thawed frozen autograft. An MRD‐negative autograft was used to prepare the dilution series. The sensitivity test revealed a strong correlation between the MRD levels of 5 × 10^−7^ and 1 × 10^−4^ (Figure 1B; *r* = 0.9997, *p* < 0.0001), indicating that NGF can accurately detect as few as 5 × 10^−7^ MM cells.

### 3.3 Comparison of MRD levels determined with NGF and NGS

MRD was assessable in autografts in 40 of 49 (82%) cases by NGS and in 49 of 49 (100%) cases by NGF. The assessment failure of NGS was thought to be due to the low quality of DNA extracted from BM aspiration slides at diagnosis, which were used to determine gene sequences unique to each patient in 43 of the 49 (88%) cases. The results of the MRD detection in 40 cases were compared between NGF and NGS. The NGS limit of detection (LOD) was 1.7 × 10^−7^ to 8.7 × 10^−5^ (median, 7 × 10^−7^) using 0.5–1 ml autografts based on the amount of DNA; the LOD of NGF was 1.2 × 10^−7^ to 5.5 × 10^−5^ (median, 7.1 × 10^−7^) using 1–10 ml autografts based on the detection of ≥10 abnormal cells and analyzed total cell number. The MRD levels determined using NGF and NGS were highly correlated (Figure 1C; *r* = 0.811, *p* < 0.0001). The MRD levels determined by the two methods were discordant in four cases; one case was MRD‐negative in NGF and MRD‐positive in NGS, and three cases were MRD‐negative in NGS and MRD‐positive in NGF.

### 3.4 PFS and OS in this cohort

**FIGURE 2:**
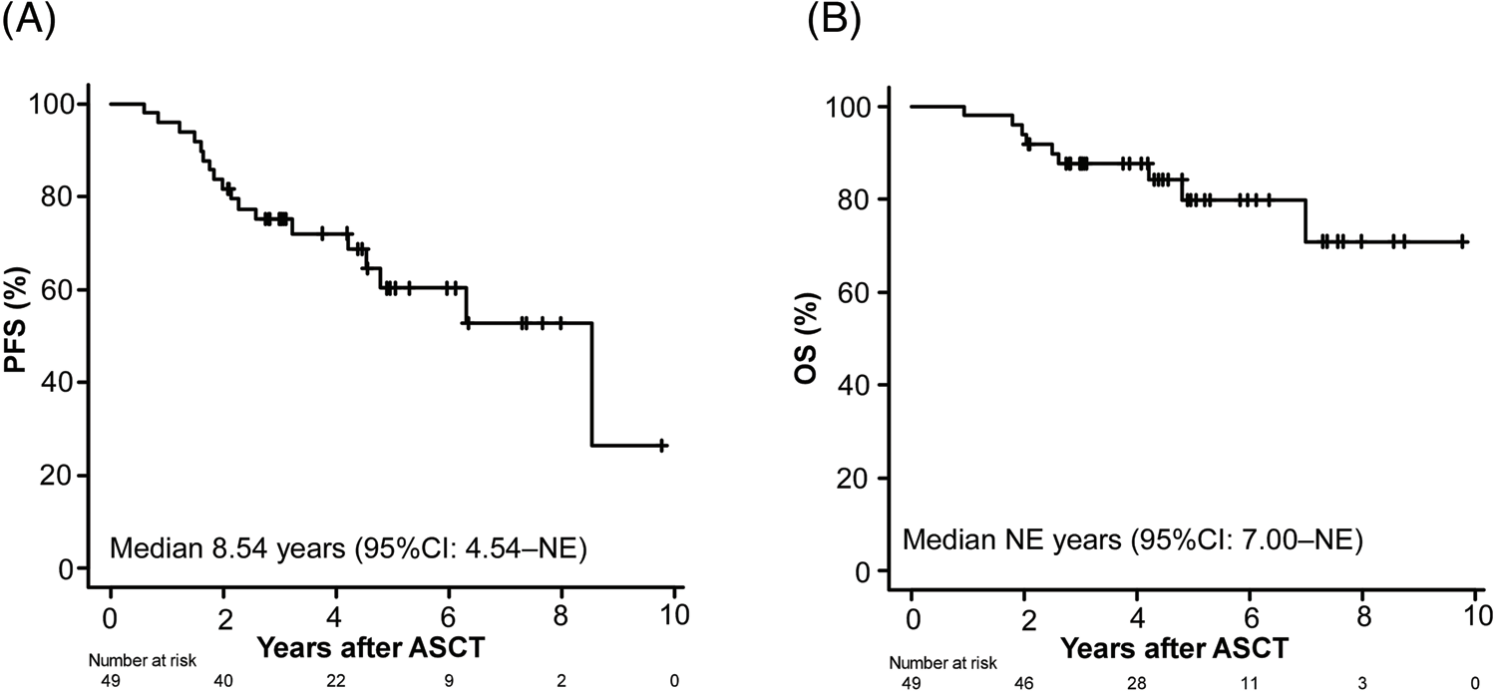
**Patient survival in this study**. (A) Progression‐free survival (PFS) and (B) overall survival (OS). ASCT, autologous stem‐cell transplantation; CI, confidence interval; NE, not estimable

Figure 2A,B shows the PFS and OS of all the patients in this cohort, respectively. With a median post‐ASCT follow‐up period of 4.3 years, the 4‐year PFS was 72% and the 4‐year OS was 88%, which is generally consistent with the results of a recent cohort study conducted in a Japanese population (*n* = 342; 3‐year PFS, 64.3%; 3‐year OS, 90.3%) [15].

### 3.5 Comparison of the prognostic value of MRD in autografts between NGF and NGS

**FIGURE 3:**
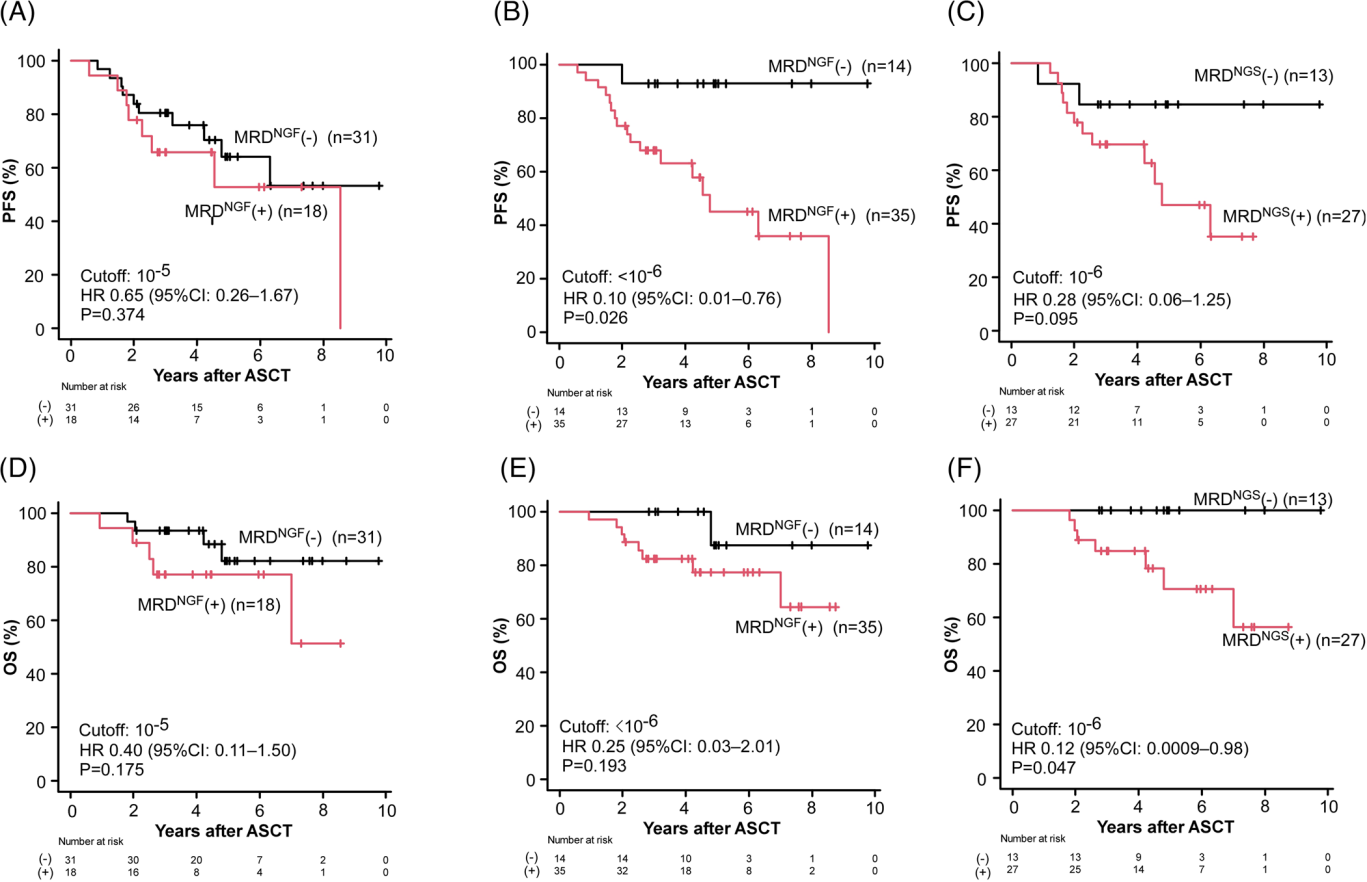
**Patient survival according to minimal/measurable residual disease (MRD)‐negativity**. (A) Progression‐free survival (PFS) determined by EuroFlow‐next generation flow (NGF) (MRD cutoff: 10^−5^) and (B) NGF (MRD cutoff: <10^−6^); (C) PFS determined by next‐generation sequencing (NGS) (MRD cutoff: 10^−6^); (D) overall survival (OS) determined by NGF (MRD cutoff: 10^−5^), (E) NGF (MRD cutoff: <10^−6^), and (F) NGS (MRD cutoff: 10^−6^). ASCT, autologous stem‐cell transplantation; CI, confidence interval; HR, hazard ratio; (+), positive; (−), negative.

As shown in Figure 1B, the detection limit of MRD by NGF increased to 5 × 10^−7^ when a sufficient number of cells were analyzed. Figure 3A,B shows PFS based on the MRD status in autografts with a cutoff MRD value of 10^−5^ and <10^−6^ (high‐sensitivity NGF), respectively. The difference in PFS between MRD‐negative and MRD‐positive cases was more evident when they were stratified according to the cutoff of MRD at <10^−6^. MRD‐negative patients identified with high‐sensitivity NGF showed significantly longer PFS than MRD‐positive patients (*p* = 0.026). PFS of the patients who were identified as MRD‐positive with high‐sensitivity NGF was similar to that of the patients identified as MRD‐negative with NGS (cutoff: 10^−6^) (Figure 3C). Figure 3D,E shows the OS of two groups divided according to the MRD status based on the sensitivity of 10^−5^ and <10^−6^, respectively. OS showed a trend similar to that of PFS based on the sensitivity of NGF and NGS (Figure 3C,F). Ten cases who were negative for MRD in both high‐sensitivity NGF and NGS showed no progression or death during the follow‐up period (Figure S1A,B), suggesting that high‐sensitivity NGF and NGS are mutually complementary in the detection of MRD. Because fewer samples were assessed by NGS (*n* = 40) compared with that by NGF (*n* = 49), PFS and OS were not statistically significantly different between MRD‐negative and MRD‐positive cases (Figure S1A,B) even though significant differences were observed in PFS by NGF and OS by NGS (Figure 3B,F).

The relationships between MRD‐negativity and pre‐ and post‐ASCT response were investigated to determine whether MRD‐negativity in autografts is a surrogate marker of a deeper response. MRD‐negativity in autografts using NGF and NGS was more achievable in patients with a deeper response pre‐ASCT, which leads to a higher CR rate (approximately 85%) post‐ASCT (Figure S2A,B). This finding suggests that MRD‐negativity in autografts is a surrogate marker of deeper response and good prognosis. The correlation between MRD levels in autografts and post‐ASCT BM (*n* = 6; median days, 99; range, 92–124) was strong (*r* = 0.976, *p* < 0.01; Figure S3), suggesting that MRD levels in autografts might predict BM post‐ASCT.

Of the 49 autografts evaluated using NGF, MRD‐negative samples were identified in 31 (63%) and 14 (29%) when the cutoff was set at 10^−5^ and <10^−6^, respectively. Three (20%) of 15 high‐risk chromosomal abnormality (HRCA) cases showed MRD levels <10^−6^ in autografts, whereas 11 (32%) of the 34 non‐HRCA cases showed the same MRD levels.

### 3.6 Univariate analysis of prognostic factors in PFS and OS

Univariate analyses were performed using known prognostic factors (Table 2). MRD negativity in autografts identified with high‐sensitivity NGF and NGS were the only prognostic factor for PFS and OS, respectively. Multivariate analyses were not used because of the small number of events in this study cohort.

**TABLE 2:** Univariate analysis for progression‐free survival and overall survival (OS)

**Factor**	**Hazard ratio for PFS (95% CI)**	***p*‐Value**	**Hazard ratio for OS (95% CI)**	***p* Value**
Female vs. male	0.79 (0.30–2.09)	0.634	1.48 (0.36–6.00)	0.584
Age ≤59 vs. ≤60	1.27 (0.50–3.23)	0.615	0.78 (0.21–2.92)	0.717
ISS I+II vs. III	1.37 (0.83–2.26)	0.220	1.61 (0.84–3.12)	0.154
HRCA (–) vs. (+)	1.67 (0.64–4.41)	0.298	1.87 (0.50–6.98)	0.353
MRD in autograft by NGF (cutoff: 10^−5^) (+) vs. (−)	0.65 (0.26–1.67)	0.374	0.40 (0.11–1.50)	0.175
MRD in autograft by NGF (cutoff: <10^−6^) (+) vs. (−)	0.10 (0.01–0.76)	0.026	0.25 (0.03–2.01)	0.193
MRD in autograft by NGS (cutoff: 10^−6^) (+) vs. (−)	0.28 (0.06–1.25)	0.095	0.12 (0.0009–0.98)	0.047

Abbreviations: HRCA, high‐risk chromosomal abnormality (*t*(4,14)/*t*(14,16)/del 17p by iFISH); ISS, International Staging System; MRD, minimal/measurable residual disease; NGF, next‐generation flow; NGS, next‐generation sequencing; PFS, progression‐free survival; (−), negative; (+), positive.

## 4 DISCUSSION

MRD negativity in autografts was considered unachievable before novel agents such as proteasome inhibitors and immunomodulatory drugs became available [16]. Therefore, Stewart et al. performed a purging using CD34^+^ selection to remove contaminated myeloma cells from autografts, thereby reducing the number of myeloma cells, although could not demonstrate improved disease‐free survival and OS possibly owing to the relatively large number of myeloma cells in patients pre‐ASCT despite receiving contamination‐free autografts. Performing autograft purging may prolong PFS and OS because of the various types of new agents. Our study demonstrated that autografts in many cases (59% and 29% when assessed by at cutoff of 10^−5^ and <10^−6^) became negative for MRD after induction therapy with those novel agents; these findings are consistent with previous reports [6, 7]. Tandem ASCT has proven effective in patients with MM who have high‐risk cytogenetic abnormalities and is being recommended as the standard therapy by the European Society for Medical Oncology guidelines [5]. The MRD‐negativity in autografts may be a reason for the success of tandem ASCT. One of the practical strategies to measure MRD levels in autografts using a highly sensitive method (cutoff value: <10^−6^) is to perform single/tandem ASCT with consolidation/maintenance therapy based on MRD levels to achieve MRD‐negative status in the whole body, including BM.

It is well‐known that NGF requires more cells than NGS to achieve the same sensitivity of MRD detection. The EuroFlow guidelines state that at least 20 cells and 50 cells must be identified as a cluster to confirm the LOD and limit of quantification of MRD, respectively [11]. Thus, more than 4 × 10^7^ cells need to be analyzed to achieve an LOD of 5 × 10^−7^, necessitating approximately 80 min/sample at a flow rate of 8000 cells/s. As shown in this study, a high sensitivity of 5 × 10^−7^ was ensured when a sufficient number of cells were available for NGF. In contrast, the same sensitivity level can be obtained using 20 μg of DNA (sensitivity of 1 × 10^−6^: 3.076 × 10^6^ cells assuming 6.5 pg/cell) and 40 μg of DNA (sensitivity of 8.6 × 10^−7^; 6.153 × 10^6^ cells) by NGS (personal communication with Adaptive Biotechnologies). Thus, the average number of cells required for NGS to achieve the same sensitivity as NGF is thought to be less than those by high‐sensitivity NGF.

One of the concerns associated with the use of cryopreserved/thawed cells is that the debris derived from dead cells might have a negative influence on MRD assessments. However, the autografts that had been cryopreserved and thawed using the conventional method with a removal of cell debris with a mesh filter–enabled accurate detection of MRD by NGF. In this study, a strong correlation was observed in the MRD levels detected between fresh and cryopreserved thawed samples from the same autografts (Figure 1A), and a high sensitivity of NGF was confirmed with samples from cryopreserved‐thawed autografts (Figure 1B). Thus, the use of cryopreserved autografts for detecting MRD is recommend when fresh samples are unavailable. We also confirmed that it is possible to analyze MRD in BM using this method (data not shown).

Because MRD levels can be greatly reduced by the development of novel agents such as proteasome inhibitors, IMiDs, monoclonal antibodies [17], BiTEs [18], and CAR‐T [19, 20], innovative high‐sensitivity MRD detection methods have become increasingly important. Conventional MRD detection methods such as NGS and NGF have been applied to BM because MM has a strong effect on BM. As a result, evidence to support the clinical significance of MRD detection in BM has accumulated [21, 22, 23]. According to the guidelines of International Myeloma Working Group, BM cells are recommended as a material to assess MRD levels, and the cutoff value is set to be 10^−5^ because of a difficulty in obtaining sufficient cells. However, the use of BM cells is associated with several problems. First, BM aspirates are often diluted by peripheral blood, which may underestimate MRD. Second, imaging studies using CT, FDG–PET/CT, and MRI have shown that MM cells patchily reside in the systemic BM. The patchy distribution of MM lesions can produce false‐negative results when a BM aspirate from limited cites is used for the analyses. Even if MRD negativity in the BM was achieved, relapses may originate from extramedullary lesions [24]. On the other hand, MRD levels in peripheral blood cells reflect the tumor burden in the whole body of patients with MM [25, 26], although only a few studies focused on the usefulness of MRD levels detected in peripheral blood, including autografts [6, 7, 20] using highly sensitive MRD detection methods.

MRD can be detected more accurately in autografts than in the BM because of the higher numbers of cells available for NGF or NGS. Nonetheless, the high sensitivity (cutoff value: <10^−6^) identified in this study has not been reported by previous studies using the EuroFlow‐NGF method. One of the major reasons for using BM instead of PB to detect MRD may be a lower detection limit when PB is used. Previous reports have shown that the MRD level in BM is 10–1000 times higher than that in PB [27]. This study showed that the detection limit of MRD could be increased up to 5 × 10^−7^ using autografts, and that MRD detection was useful for predicting patient outcome. In addition to the aforementioned use of autografts, serum can be used to assess MRD using mass spectrometry [28, 29, 30]. This technology may be as sensitive as NGF and NGS for the detection of MRD.

To date, several studies have compared the value of MRD in BM or autografts in the prediction of PFS and OS between NGF and NGS [8, 17, 31]. In general, the sensitivity of NGS was higher than that of NGF when the same number of cells was subjected to the analyses. However, if the sensitivity to detect MRD is the same between NGS and NGF, the two methods have the same ability to predict PFS and OS [17, 31]. The results of our study validate this hypothesis.

This study had some limitations. First, we retrospectively analyzed a relatively small number of patients who received nonuniform treatment. Second, the R‐ISS data for statistical analysis could not be obtained because of the lack of lactate dehydrogenase data. Third, due to the retrospective nature of the analysis, we were unable to assess MRD levels in BM of all patients concurrently with their autografts.

In conclusion, the modified high‐sensitivity EuroFlow‐NGF method can be used to assess MRD in frozen/thawed autografts and increase the sensitivity for detection of MRD to a level of 5 × 10^−7^, which is comparable to that of NGS. The NGF is as useful as NGS for predicting the prognosis of patients with MM. Large‐scale prospective studies are warranted to confirm these results.

### AUTHOR CONTRIBUTIONS

Ryota Urushihara, Naoki Takezako, Takeshi Yoroidaka, Takeshi Yamashita, Ryoichi Murata, Shinji Nakao, and Hiroyuki Takamatsu collected clinical data and clinical samples. Ryota Urushihara and Hiroyuki Takamatsu performed flow cytometry and analyzed data. Kenji Satou helped statistical analysis and analyzed data. Hiroyuki Takamatsu designed the research. Ryota Urushihara, Shinji Nakao, and Hiroyuki Takamatsu wrote the manuscript. All authors critically reviewed the manuscript and checked the final version.

## CONFLICT OF INTEREST

H.T. received honoraria from Janssen Pharmaceutical K.K., Bristol‐Myers Squibb company, Sanofi, Takeda Pharmaceutical Company Limited and Ono Pharmaceutical Co., Ltd. T. Yamashita received honoraria from Bristol‐Myers Squibb company, Sanofi, Takeda Pharmaceutical Company Limited and Ono Pharmaceutical Co., Ltd. The rest of authors do not have any relationships to disclose.

## ETHICS STATEMENT

All patients provided written informed consent in accordance with the Declaration of Helsinki. This study was approved by the Institutional Review Board of Kanazawa University (No. 2015‐231) and each hospital.

## Supporting information

Supporting Information

Supporting Figures

## Data Availability

The data that support the findings of this study are available from the corresponding author upon reasonable request.

## References

[1] Palumbo A , Cavallo F , Gay F , Di Raimondo F , Ben Yehuda D , Petrucci MT , et al. Autologous transplantation and maintenance therapy in multiple myeloma. N Engl J Med. 2014;371(10):895–905. 25184862 10.1056/NEJMoa1402888

[2] Attal M , Lauwers‐Cances V , Hulin C , Leleu X , Caillot D , Escoffre M , et al. Lenalidomide, bortezomib, and dexamethasone with transplantation for myeloma. N Engl J Med. 2017;376(14):1311–20. 28379796 10.1056/NEJMoa1611750 PMC6201242

[3] Cavo M , Gay F , Beksac M , Pantani L , Petrucci MT , Dimopoulos MA , et al. Autologous haematopoietic stem‐cell transplantation versus bortezomib‐melphalan‐prednisone, with or without bortezomib‐lenalidomide‐dexamethasone consolidation therapy, and lenalidomide maintenance for newly diagnosed multiple myeloma (EMN02/HO95): a multicentre, randomised, open‐label, phase 3 study. Lancet Haematol. 2020;7(6):e456–68. 32359506 10.1016/S2352-3026(20)30099-5

[4] Gay F , Musto P , Rota‐Scalabrini D , Bertamini L , Belotti A , Galli M , et al. Carfilzomib with cyclophosphamide and dexamethasone or lenalidomide and dexamethasone plus autologous transplantation or carfilzomib plus lenalidomide and dexamethasone, followed by maintenance with carfilzomib plus lenalidomide or lenalidomide alone for patients with newly diagnosed multiple myeloma (FORTE): a randomised, open‐label, phase 2 trial. Lancet Oncol. 2021;22(12):1705–20. 34774221 10.1016/S1470-2045(21)00535-0

[5] Dimopoulos MA , Moreau P , Terpos E , Mateos MV , Zweegman S , Cook G , et al. Multiple myeloma: EHA‐ESMO Clinical Practice Guidelines for diagnosis, treatment and follow‐up(dagger). Ann Oncol. 2021;32(3):309–22. 33549387 10.1016/j.annonc.2020.11.014

[6] Bal S , Landau HJ , Shah GL , Scordo M , Dahi P , Lahoud OB , et al. Stem cell mobilization and autograft minimal residual disease negativity with novel induction regimens in multiple myeloma. Biol Blood Marrow Transplant. 2020;26(8):1394–401. 32442725 10.1016/j.bbmt.2020.04.011 PMC7371503

[7] Takamatsu H , Takezako N , Zheng J , Moorhead M , Carlton VEH , Kong KA , et al. Prognostic value of sequencing‐based minimal residual disease detection in patients with multiple myeloma who underwent autologous stem‐cell transplantation. Ann Oncol. 2017;28(10):2503–10. 28945825 10.1093/annonc/mdx340 PMC5834061

[8] Takamatsu H , Takezako N , Wee RK , Yoroitaka T , Yamashita T , Murata R , et al. Comparison of MRD detection in autografts of patients with multiple myeloma between 8‐color MFC (EuroFlow‐NGF) and NGS. Blood. 2018;132:258.

[9] Takamatsu H , Takezako N , Yoroidaka T , Yamashita T , Murata R , Yamazaki A , et al. Minimal residual disease in autografts and bone marrow of patients with multiple myeloma: 8‐color multiparameter flow cytometry (EuroFlow‐NGF) vs. next‐generation sequencing. Blood. 2020;136:3174.

[10] Ching T , Duncan ME , Newman‐Eerkes T , McWhorter MME , Tracy JM , Steen MS , et al. Analytical evaluation of the clonoSEQ Assay for establishing measurable (minimal) residual disease in acute lymphoblastic leukemia, chronic lymphocytic leukemia, and multiple myeloma. BMC Cancer. 2020;20(1):612. 32605647 10.1186/s12885-020-07077-9 PMC7325652

[11] Flores‐Montero J , Sanoja‐Flores L , Paiva B , Puig N , Garcia‐Sanchez O , Bottcher S , et al. Next generation flow for highly sensitive and standardized detection of minimal residual disease in multiple myeloma. Leukemia. 2017;31(10):2094–103. 28104919 10.1038/leu.2017.29 PMC5629369

[12] Durie BG , Harousseau JL , Miguel JS , Blade J , Barlogie B , Anderson K , et al. International uniform response criteria for multiple myeloma. Leukemia. 2006;20(9):1467–73. 16855634 10.1038/sj.leu.2404284

[13] Heinze G , Schemper M . A solution to the problem of monotone likelihood in Cox regression. Biometrics. 2001;57(1):114–9. 11252585 10.1111/j.0006-341x.2001.00114.x

[14] Kanda Y . Investigation of the freely available easy‐to‐use software ‘EZR’ for medical statistics. Bone Marrow Transplant. 2013;48(3):452–8. 23208313 10.1038/bmt.2012.244 PMC3590441

[15] Shibayama H , Itagaki M , Handa H , Yokoyama A , Saito A , Kosugi S , et al. Primary survival analysis of Japanese patients with plasma cell neoplasms in novel drugs era. The 84th Japanese Society of Hematology annual meeting, abstract #OS1‐12D‐1. 2022.

[16] Stewart AK , Vescio R , Schiller G , Ballester O , Noga S , Rugo H , et al. Purging of autologous peripheral‐blood stem cells using CD34 selection does not improve overall or progression‐free survival after high‐dose chemotherapy for multiple myeloma: results of a multicenter randomized controlled trial. J Clin Oncol. 2001;19(17):3771–9. 11533101 10.1200/JCO.2001.19.17.3771

[17] Medina A , Puig N , Flores‐Montero J , Jimenez C , Sarasquete ME , Garcia‐Alvarez M , et al. Comparison of next‐generation sequencing (NGS) and next‐generation flow (NGF) for minimal residual disease (MRD) assessment in multiple myeloma. Blood Cancer J. 2020;10(10):108. 33127891 10.1038/s41408-020-00377-0 PMC7603393

[18] Moreau P , Garfall AL , van de Donk N , Nahi H , San‐Miguel JF , Oriol A , et al. Teclistamab in relapsed or refractory multiple myeloma. N Engl J Med. 2022;387(6):495–505. 35661166 10.1056/NEJMoa2203478 PMC10587778

[19] Raje N , Berdeja J , Lin Y , Siegel D , Jagannath S , Madduri D , et al. Anti‐BCMA CAR T‐cell therapy bb2121 in relapsed or refractory multiple myeloma. N Engl J Med. 2019;380(18):1726–37. 31042825 10.1056/NEJMoa1817226 PMC8202968

[20] Cohen AD , Mateos MV , Cohen YC , Rodriguez‐Otero P , Paiva B , van de Donk N , et al. Efficacy and safety of cilta‐cel in patients with progressive MM after exposure to other BCMA‐targeting agents. Blood. 2022 Sep 12;blood.2022015526. 10.1182/blood.2022015526. Online ahead of print. PMC10562529 36095849

[21] Costa LJ , Chhabra S , Medvedova E , Dholaria BR , Schmidt TM , Godby KN , et al. Daratumumab, carfilzomib, lenalidomide, and dexamethasone with minimal residual disease response‐adapted therapy in newly diagnosed multiple myeloma. J Clin Oncol. 2022;40:2901–12. 34898239 10.1200/JCO.21.01935

[22] Richardson PG , Jacobus SJ , Weller EA , Hassoun H , Lonial S , Raje NS , et al. Triplet therapy, transplantation, and maintenance until progression in myeloma. N Engl J Med. 2022;387(2):132–47. 35660812 10.1056/NEJMoa2204925 PMC10040899

[23] Paiva B , Manrique I , Dimopoulos MA , Gay F , Min CK , Zweegman S , et al. MRD dynamics during maintenance for improved prognostication of 1280 myeloma patients in TOURMALINE‐MM3 and ‐MM4 trials. Blood. 2022.10.1182/blood.202201678236130300

[24] Paiva B , Puig N , Cedena MT , Cordon L , Vidriales MB , Burgos L , et al. Impact of next‐generation flow (NGF) minimal residual disease (MRD) monitoring in multiple myeloma (MM): results from the Pethema/GEM2012 Trial. Blood. 2017;130:905.

[25] Sanoja‐Flores L , Flores‐Montero J , Puig N , Contreras‐Sanfeliciano T , Pontes R , Corral‐Mateos A , et al. Blood monitoring of circulating tumor plasma cells by next generation flow in multiple myeloma after therapy. Blood. 2019;134(24):2218–22. 31697808 10.1182/blood.2019002610 PMC6966491

[26] Bertamini L , Oliva S , Rota‐Scalabrini D , Paris L , More S , Corradini P , et al. High levels of circulating tumor plasma cells as a key hallmark of aggressive disease in transplant‐eligible patients with newly diagnosed multiple myeloma. J Clin Oncol. 2022;40(27):3120–31. 35666982 10.1200/JCO.21.01393

[27] Vij R , Mazumder A , Klinger M , O'Dea D , Paasch J , Martin T , et al. Deep sequencing reveals myeloma cells in peripheral blood in majority of multiple myeloma patients. Clin Lymphoma Myeloma Leuk. 2014;14(2):131–9.e1. 24629890 10.1016/j.clml.2013.09.013

[28] Abeykoon JP , Murray DL , Murray I , Jevremovic D , Otteson GE , Dispenzieri A , et al. Implications of detecting serum monoclonal protein by MASS‐fix following stem cell transplantation in multiple myeloma. Br J Haematol. 2021;193(2):380–5. 33216966 10.1111/bjh.17195

[29] Murray DL , Puig N , Kristinsson S , Usmani SZ , Dispenzieri A , Bianchi G , et al. Mass spectrometry for the evaluation of monoclonal proteins in multiple myeloma and related disorders: an International Myeloma Working Group Mass Spectrometry Committee Report. Blood Cancer J. 2021;11(2):24. 33563895 10.1038/s41408-021-00408-4 PMC7873248

[30] Dispenzieri A , Krishnan A , Arendt B , Blackwell B , Wallace PK , Dasari S , et al. Mass‐fix better predicts for PFS and OS than standard methods among multiple myeloma patients participating on the STAMINA trial (BMT CTN 0702/07LT). Blood Cancer J. 2022;12(2):27. 35145071 10.1038/s41408-022-00624-6 PMC8831597

[31] Oliva S , Genuardi E , Petrucci MT , D'Agostino M , Auclair D , Spadano A , et al. Impact of minimal residual disease (MRD) by multiparameter flow cytometry (MFC) and next‐generation sequencing (NGS) on outcome: results of newly diagnosed transplant‐eligible multiple myeloma (MM) patients enrolled in the forte trial. Blood. 2020;136:44–5.

