# Supplementary material for: Eight‐color multiparameter flow cytometry (EuroFlow‐NGF) is as sensitive as next‐generation sequencing in detecting minimal/measurable residual disease in autografts of patients with multiple myeloma
Source: EJHaem. 2023 Jan 24;4(1):184–91. doi: 10.1002/jha2.633 (PMC9928796; doi:10.1002/jha2.633)
Supplement: Supplementary file 1 — Supporting Information [file JHA2-4-184-s002.docx]

**Supplement:**

**Method of high-sensitivity next-generation flow (NGF) based on the protocol of the EuroFlow Bulk Lysis protocol for MRD panels (version 1.1** [**May 6, 2014**]**) and the reference of CYT-MM-MRD (Cytognos, Salamanca, Spain).**

Typically, 300 µL of thawed cell suspension should remain in the tube.

2.5. Add 2 mL of PBS + 0.09% of NaN3 + 0.5 % of BSA and resuspend the cell pellet vigorously.

2.6. Complete the volume of the tube containing the cell suspension up to 50 mL final volume with PBS + 0.09% of NaN3 + 0.5 % of BSA.

2.7. Mix well.

2.8. Centrifuge at 800 g for 5 min and remove the supernatant using a Pasteur pipette without disturbing the cell pellet.

2.9. Resuspend the cell pellet in 2 mL of PBS + 0.09% of NaN3 + 0.5 % of BSA. Mix well and transfer this volume to a 5 mL polystyrene round‐bottom Falcon tube (“FACS tube”).

2.10. Wash the 50 mL Falcon tube with 2 mL of PBS + 0.09% of NaN3 + 0.5 % of BSA more to recover cells that might have been left in the original tube. Add this volume to the 5 mL Falcon tube containing the rest of the sample transferred in step 2.9.

2.11. Centrifuge at 540 g for 5 min and remove the supernatant using a Pasteur pipette. If the remaining cell volume is lower than 300 µL, PBS + 0.09% of NaN3 + 0.5 % of BSA will be added to reach a volume of at least 300 µL.

2.12. In case multiple 50 mL tubes were used (because it was needed to lyse large sample volumes) the cell suspensions from the same sample should be combined at this moment, before adjusting cell concentration. Try to keep the final volume low, so that, in case that cell concentration needs to be adjusted as indicated in the next step, it can be easily done by diluting with the recommended buffer.

2.13. Adjust the final cells concentration to approximately 0.5 x 10^5^–4 x 10^5^ cells /μL, by resuspending the pellet with PBS + 0.09% of NaN3 + 0.5 % of BSA.

2.14. Calculate 200 μL (i.e. 10-80 million cells) of the sample solution per each tube to be stained/acquired.

**Staining steps for surface membrane markers only (MM-MRD Tube 1 [Table S1])**

1. Reconstitute the lyophilized vial following steps in “Reconstituting the Lyophilized Reagents” section.

2. Add 30 μL of reconstituted antibody mixture in a 5 mL flow cytometry tube.

3. Add 10μL of CD27 BV510 and 2μL of CD138 BV421 to the tube.

4. Add 100 μL of cell suspension containing 5-40 x 10^6^ cells to the tube.

5. Mix well by vortexing.

6. Bring the final volume to 200 μL with wash buffer.

7. Incubate for 30 minutes at RT protected from light.

8. Add 2 mL of 1X BD FACS™ Lysing Solution.

9. Mix well by vortexing.

10. Incubate for 10 minutes at RT protected from light.

11. Centrifuge for 5 minutes at 540*g*.

12. Discard the supernatant using a Pasteur pipette without disturbing the cell pellet, leaving approximately 100 μL of residual volume in the tube.

13. Add 2 mL of wash buffer.

14. Mix well by vortexing.

15. Centrifuge for 5 minutes at 540*g*.

16. Discard the supernatant using a Pasteur pipette without disturbing the cell pellet, leaving approximately 100 μL of residual volume in the 5 mL tube.

17. Resuspend the cell pellet with acquisition buffer in a total volume of 500 μL.

18. Acquire the cells immediately after staining or store at 4°C (for 1 hour maximum) until acquisition.

**Staining steps for combined staining of surface membrane and cytoplasmic markers (MM-MRD Tube 2 [Table S1])**

1. Reconstitute the lyophilized vial for surface staining following steps in “Reconstituting the Lyophilized Reagents” section.

2. Add 20 μL of reconstituted surface staining antibody mixture to a 5 mL flow cytometry tube.

3. Add 10μL of CD27 BV510 and 2μL of CD138 BV421 to the tube.

4. Add 100 μL of cell suspension containing 5-40 x 10^6^ cells to the tube.

5. Mix well by vortexing.

6. Bring the final volume to 200 μL with wash buffer.

7. Incubate for 30 minutes at RT protected from light.

8. Add 2 mL of wash buffer to the tube containing the cell pellet.

9. Mix well by vortexing.

10. Centrifuge for 5 minutes at 540*g*.

11. Discard the supernatant using a Pasteur pipette without disturbing the cell pellet, leaving approximately 100 μL of residual volume in the tube.

12. Resuspend the cell pellet by mixing gently.

13. Add 100 μL of Fix&Perm® Solution A (Fix&Perm®, Nordic-MUBio BV, The Netherlands) and mix thoroughly by vortexing for 1-2 seconds.

14. Incubate for 15 minutes at RT protected from light.

15. Add 2 mL of wash buffer to the tube containing the cell pellet.

16. Mix well by vortexing.

17. Centrifuge for 5 minutes at 540*g*.

18. Discard the supernatant using a Pasteur pipette without disturbing the cell pellet, leaving approximately 100 μL residual volume in the 5 mL tube.

19. Vortex vigorously to resuspend the cell pellet.

20. Add 100 μL of Fix&Perm® Solution B (Fix&Perm®, Nordic-MUBio BV, The Netherlands).

21. Mix well by vortexing.

22. Reconstitute the lyophilized vial for cytoplasmic staining following steps in “Reconstituting the Lyophilized Reagents” section.

23. Add 10 μL of reconstituted cytoplasmic staining antibody mixture to the tube containing the 200 μL of cell suspension in Fix&Perm® Solution B (Fix&Perm®, Nordic-MUBio BV, The Netherlands) and cell suspension.

24. Mix well by vortexing.

25. Incubate for 15 minutes at RT protected from light.

26. Add 2 mL of wash buffer to the tube containing the cell pellet.

27. Mix well by vortexing.

28. Centrifuge for 5 minutes at 540*g*.

29. Discard the supernatant using a Pasteur pipette without disturbing the cell pellet, leaving approximately 100 μL of residual volume in the tube.

30. Resuspend the cell pellet with acquisition buffer in a total volume of 500 μL.

31. Acquire the cells immediately after staining or store at 4°C (for 1 hour maximum) until acquisition.

Acquire tube completely at a flow rate adjusted up to 8000 cells/sec.

The FACSCanto^TM^ II (BD Biosciences, Franklin Lakes, NJ, USA) flow cytometer was used to measure all samples, and gating and identification of clonal abnormal plasma cells were manually performed by experts using the Infinicyt software (Cytognos,
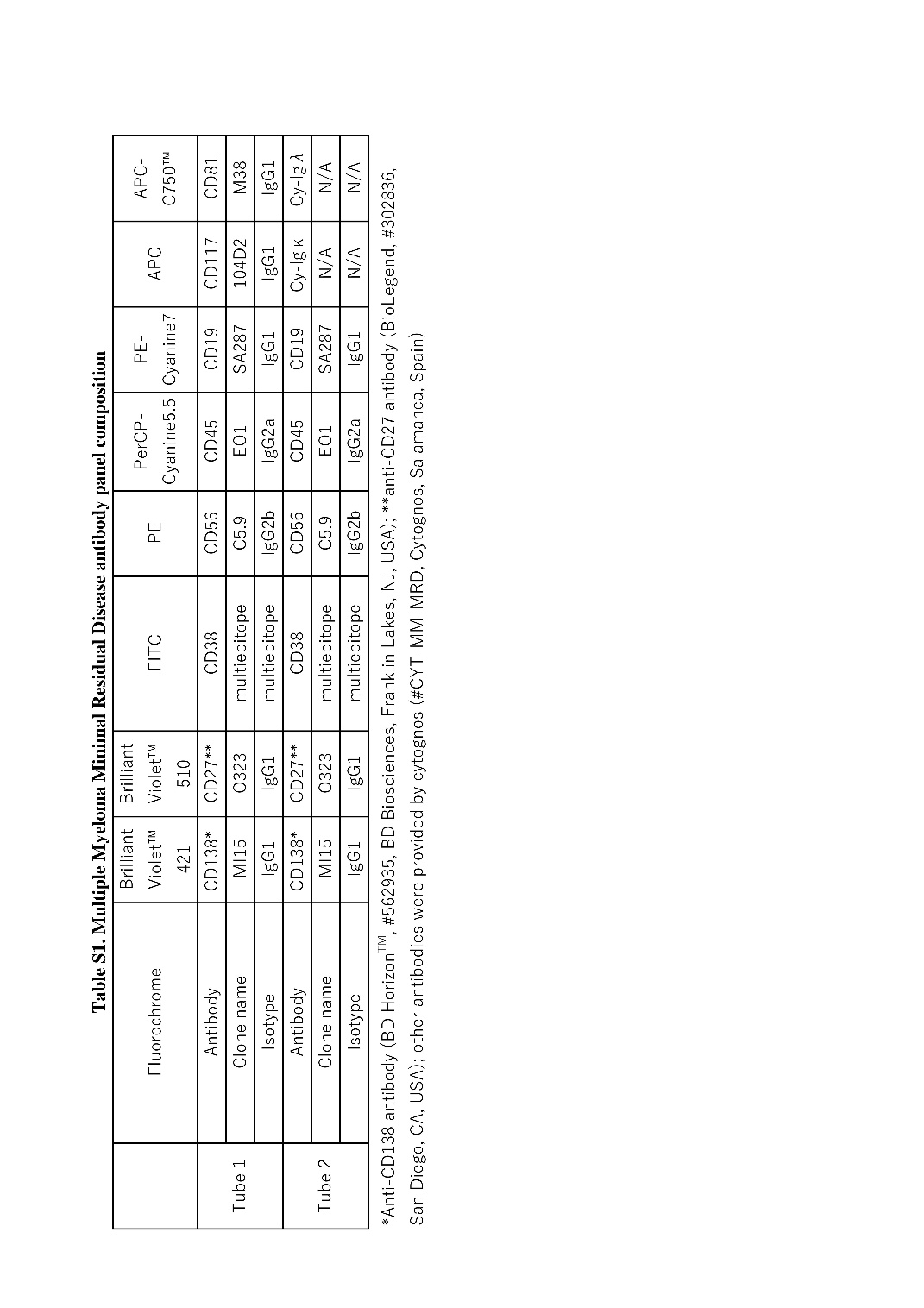
Salamanca, Spain).
