# Supplementary material for: Eight‐color multiparameter flow cytometry (EuroFlow‐NGF) is as sensitive as next‐generation sequencing in detecting minimal/measurable residual disease in autografts of patients with multiple myeloma
Source: EJHaem. 2023 Jan 24;4(1):184–91. doi: 10.1002/jha2.633 (PMC9928796; doi:10.1002/jha2.633)
Supplement: Supplementary file 2 — Supporting Figures [file JHA2-4-184-s001.pdf]

# Figure S1

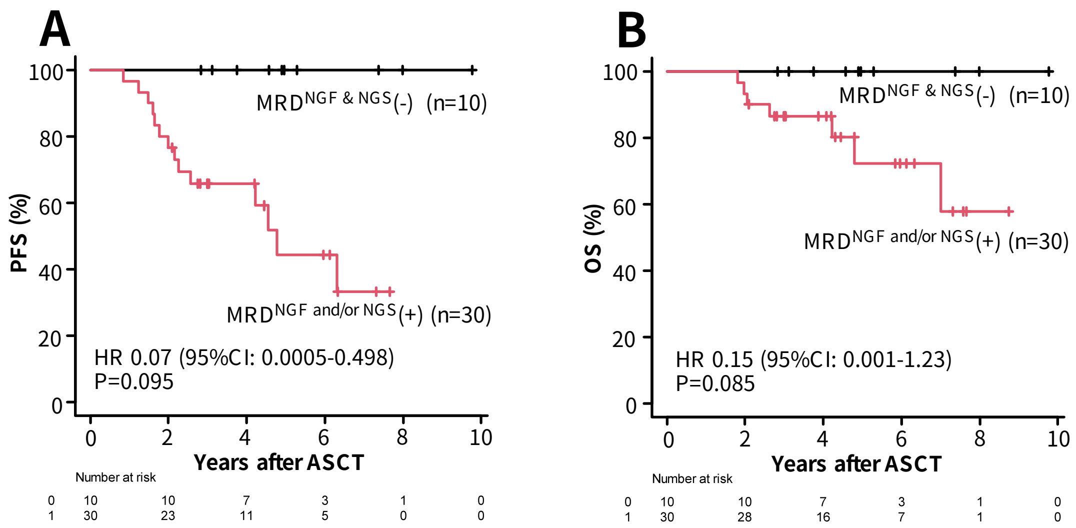


Figure S1. Survival of patients according to MRD-negativity. (A) Progression-free survival (PFS) by EuroFlow-next -generation flow (NGF) (MRD cutoff: < $10^{-6}$) and next-generation sequencing (NGS) (MRD cutoff: $10^{-6}$), (B) Overall survival (OS) by EuroFlow-next-generation flow (NGF) (MRD cutoff: < $10^{-6}$) and next-generation sequencing (NGS) (MRD cutoff: $10^{-6}$). +: positive, -: negative, HR: hazard ratio, CI: confidence interval,

Figure S2

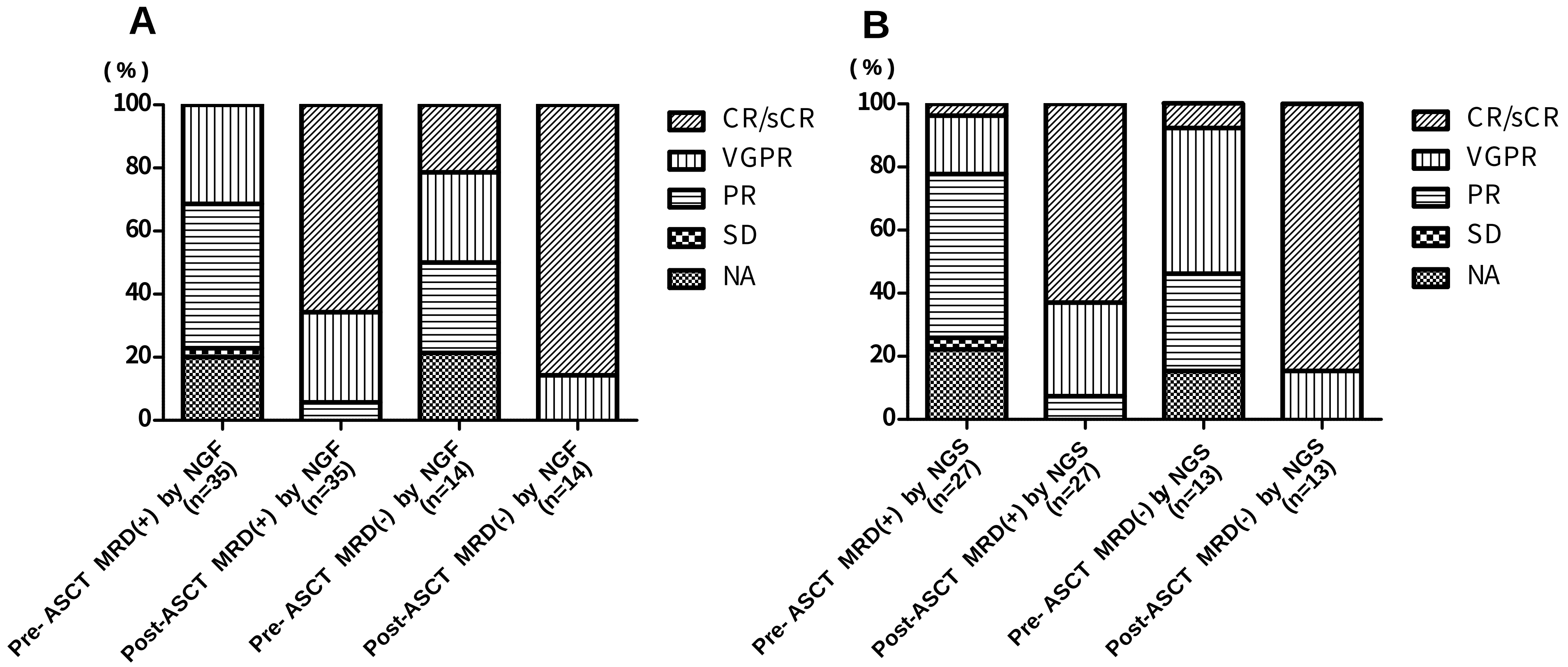


**Figure S2.** Pre and Post-ASCT response rate according to MRD-negativity (A) by EuroFlow-next-generation flow (NGF) (MRD cutoff: $< 10^{-6}$) and (B) by next-generation sequencing (NGS) (MRD cutoff: $10^{-6}$). MRD: minimal /measurable residual disease, (+): positive, (-): negative, ASCT: autologous stem cell transplantation, sCR: stringent complete response, CR: complete response, VGPR: very good partial response, PR: partial response, SD: stable disease, NA: not assessed.

Figure S3

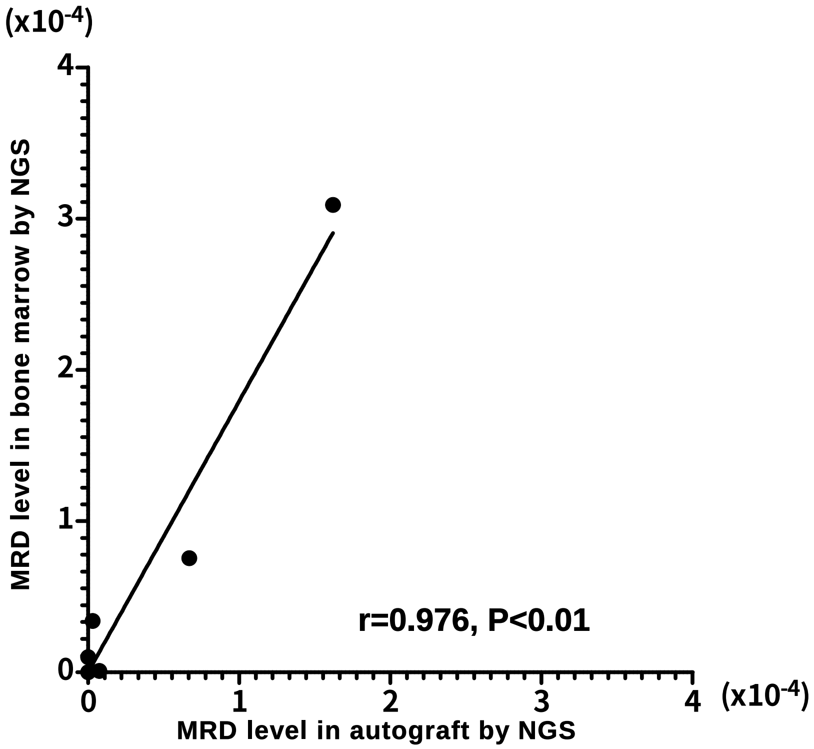


**Supplementary Figure S3. Correlation between minimal residual /measurable disease (MRD) levels in post-ASCT bone marrow (BM) (n=6; median days, 99; range, 92-124) and autograft samples (n=6) by next -generation sequencing (NGS). Correlation between MRD levels in BM and autograft samples (n=6) by NGS was analyzed using Pearson's coefficient test.**
